# Gas-Pressurized Torrefaction of Lignocellulosic Solid Wastes: Deoxygenation and Aromatization Mechanisms of Cellulose

**DOI:** 10.3390/molecules28227671

**Published:** 2023-11-20

**Authors:** Liu Shi, Yiming Sun, Xian Li, Shuo Li, Bing Peng, Zhenzhong Hu, Hongyun Hu, Guangqian Luo, Hong Yao

**Affiliations:** State Key Laboratory of Coal Combustion, School of Energy and Power Engineering, Huazhong University of Science and Technology, Wuhan 430074, China; liu_shi@hust.edu.cn (L.S.);

**Keywords:** lignocellulosic solid wastes, cellulose, gas-pressurized torrefaction, molecular structure, deoxygenation mechanism, aromatization mechanism

## Abstract

A novel gas-pressurized (GP) torrefaction method at 250 °C has recently been developed that realizes the deep decomposition of cellulose in lignocellulosic solid wastes (LSW) to as high as 90% through deoxygenation and aromatization reactions. However, the deoxygenation and aromatization mechanisms are currently unclear. In this work, these mechanisms were studied through a developed molecular structure calculation method and the GP torrefaction of pure cellulose. The results demonstrate that GP torrefaction at 250 °C causes 47 wt.% of mass loss and 72 wt.% of O removal for cellulose, while traditional torrefaction at atmospheric pressure has almost no impact on cellulose decomposition. The GP-torrefied cellulose is determined to be composed of an aromatic furans nucleus with branch aliphatic C through conventional characterization. A molecular structure calculation method and its principles were developed for further investigation of molecular-level mechanisms. It was found 2-ring furans aromatic compound intermediate is formed by intra- and inter-molecular dehydroxylation reactions of amorphous cellulose, and the removal of O-containing function groups is mainly through the production of H_2_O. The three-ring furans aromatic compound intermediate and GP-torrefied cellulose are further formed through the polymerization reaction, which enhances the removal of ketones and aldehydes function groups in intermediate torrefied cellulose and form gaseous CO and O-containing organic molecules. A deoxygenation and aromatization mechanism model was developed based on the above investigation. This work provides theoretical guidance for the optimization of the gas-pressurized torrefaction method and a study method for the determination of molecular-level structure and the mechanism investigation of the thermal conversion processes of LSW.

## 1. Introduction

Lignocellulosic solid wastes (LSW) are considered a valuable recyclable energy resource that can satisfy the growing energy demand and mitigate environmental concerns [1,2]. However, its intrinsic deficiencies, such as high water content, high oxygen content, low energy density, and poor grindability, have limited its applications as fuel [3,4]. One of the main underlying reasons for these deficiencies is ascribed to the abundant O-containing functional groups in LSW [5,6].

Torrefaction is one of the most widely used methods for deoxygenating LSW and improving their fuel qualities [7,8]. Partial unstable O-containing functional groups from LSW are effectively removed at 200–300 °C during torrefaction [3,4]. The main oxygenates in LSW are hemicellulose and cellulose [9]. Consequently, the main target of torrefaction is to deoxygenate hemicellulose and cellulose. Ma [10] reported that the O content of hemicellulose was reduced from 54.92 wt.% of raw hemicellulose to 46.35 wt.% and 38.36 wt.% through traditional torrefaction at atmospheric pressure (AP) at 240 and 300 °C. Contrastively, the O content of cellulose was slightly decreased from 52.11 wt.% of raw cellulose to 51.10 wt.% after AP torrefaction at 240 °C, which was still higher than 48 wt.% after AP torrefaction even at 300 °C. Moreover, Larachi [11] reported that the O/C ratios of torrefied hemicellulose and cellulose were decreased from 0.99 and 0.89 of raw hemicellulose and cellulose, respectively, to 0.44 and 0.83 after AP torrefaction at 280 °C. These indicate that hemicellulose is pyrolyzed easily, but cellulose is hard to be pyrolyzed by AP torrefaction at 200–300 °C. The fuel property of AP-torrefied hemicellulose is proximate to that of peat, whereas the fuel property of AP-torrefied cellulose is still proximate to that of biomass-like fuels [10,12]. It is concluded that traditional AP torrefaction has efficiency for hemicellulose deoxygenation but is limited for cellulose. However, the proportion of cellulose in LSW is generally higher than the proportion of hemicellulose and lignin [13]. These cause AP torrefaction to have limited efficiency for LSW deoxygenation. Thus, the enhancement of deoxygenation efficiency for cellulose is the key to LSW deoxygenation via torrefaction.

The limited deoxygenation efficiency of AP torrefaction for cellulose is due to its thermochemical principle. It was found that the organic functional group composition and the skeleton of cellulose have almost no change after AP torrefaction at 200–300 °C [12]. The crystallinity index (CrI) of cellulose torrefied at 250 °C was still rather similar to that of the raw cellulose and just decreased slightly after torrefaction at 300 °C [12]. Additionally, it was found that the mass loss of cellulose after AP torrefaction at 250 °C and 60 min was less than 3% [14]. Agarwal [15] found that the depolymerization of cellulose to ‘intermediate cellulose’ is the critical reaction for cellulose decomposition. Meanwhile, the Broido–Shafizadeh (B–S) model confirmed that the conversion of cellulose to ‘active cellulose’ is the main thermal decomposition reaction during AP torrefaction at 200–300 °C [16]. Furthermore, Wang found that the ‘active cellulose’ is composed of carbohydrates, such as oligosaccharides, glucose, and levoglucosan [17], indicating that oxygen was not removed after AP torrefaction at 200–300 °C. Thus, AP torrefaction has a limited efficiency for the thermal decomposition and deoxygenation of cellulose.

To improve the O removal efficiency of cellulose from LSW, various methods have been developed. The most widely used methods are wet torrefaction [18,19,20], superheated steam (SHS) torrefaction [21,22], and torrefaction at higher temperatures. The O content of cellulose was reduced from 49.4 wt.% to 23.1–24.8 wt.% via wet torrefaction at 250 °C, 3.97 MPa, and a holding time of 2–4 h [20]. Cellulose begins to thermally decompose at 200 °C during wet torrefaction [19], and it undergoes two-step reactions [23]. SHS torrefaction causes cellulose depolymerization and over 5 wt.% cellulose loss at 250 °C and a holding time of 2 h [24]. The O content of wood shaving was significantly reduced via SHS torrefaction from 48.6 wt.% to 46.9 wt.% at 250 °C with a holding time of 2 h [21]. Although these methods have a higher O removal efficiency than traditional AP torrefaction, their harsh conditions and high energy consumption prevent their industrialization.

In recent years, a novel gas-pressurized (GP) torrefaction method has been proposed, which realizes the deeper deoxidation of LSW under milder conditions [25,26,27]. GP torrefaction is conducted in a closed reactor, and the secondary reactions between LSW and volatiles occur. Furthermore, the volatiles raise the gas phase pressure in the reactor, which further enhances the secondary reactions greatly [28]. The O content of GP-torrefied LSW was as low as 21.0 wt.%, significantly lower than that obtained through AP torrefaction under the same temperature of 250 °C [29,30]. The moisture adsorption and spontaneous ignition characteristics of LSW are also significantly inhibited, and the calorific value and grindability characteristics are considerably improved via GP torrefaction. The fuel properties of GP-torrefied LSW are proximate to those of coal [30]. Detailed analysis revealed that over 90% of cellulose in LSW decomposed during GP torrefaction at 250 °C, and its CrI decreased from 37.2% of raw LSW to 18.8% of GP-torrefied LSW [29].

As explained above, the main reason for the deoxygenation efficiency enhancement of GP torrefaction is the promotion of the decomposition and deoxygenation of cellulose, which is the fundamental difference from AP torrefaction. However, the mechanism of decomposition and deoxygenation of cellulose during GP torrefaction, which is rather meaningful for the mechanistic understanding and process optimization of GP torrefaction, is scant. Thus, the objective of this work is to investigate the decomposition and deoxygenation mechanism of cellulose during GP torrefaction. Molecular structure models of intermediate and torrefied cellulose obtained by GP torrefaction were established through a molecular structure calculation method, and the reaction mechanism model of cellulose during the GP torrefaction was, thus, developed. This study provides guidance for a deeper understanding of the reaction mechanism and method optimization for GP torrefaction.

## 2. Results

### 2.1. Yield Distribution

**Figure 1:**
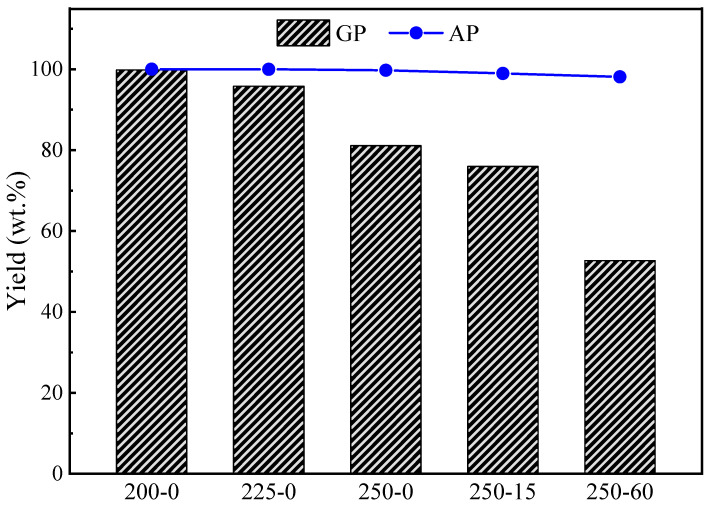
Mass yields of cellulose torrefied by GP and AP torrefactions.

Figure 1 illustrates the solid yields of GP and AP torrefactions at different temperatures and holding times. The cellulose had no obvious decomposition during AP torrefaction at 200 °C and 225 °C and GP torrefactions at 200 °C. But, the solid yields of GP-225-0 were 95.8 wt.%, implying the cellulose begins to thermally decompose during GP torrefaction at temperatures as low as 225 °C. At the same time, it remains intact during AP torrefaction under the same temperature. The solid yield decreased further with the temperature rise from 95.8 wt.% at 225 °C to 81.0 wt.% at 250 °C during the GP torrefaction, which was significantly lower than that of AP torrefaction of 99.7 wt.% at 250 °C. These clearly show that the cellulose largely decomposed during GP torrefaction at 250 °C. The yield of AP-torrefied cellulose slightly decreased with the holding time extension at 250 °C, reaching the lowest yield of 98.1 wt.% at 60 min. For the GP torrefaction at 250 °C, the yield decreased further with the holding time extension from 81.0 wt.% at 0 min to as low as 52.7 wt.% at 60 min. This significant difference in solid yields confirms that the thermal decomposition process and reaction mechanism are different from the AP and GP torrefactions.

### 2.2. Ultimate Composition of Torrefied Cellulose

#### 2.2.1. Elemental Composition

Table 1 illustrates the elemental composition of raw, GP- and AP-torrefied cellulose. The O contents of AP-torrefied cellulose had almost no change, from 51.1 wt.% of raw cellulose to 50.4 wt.% of AP-250-60 cellulose. It is confirmed that the AP torrefaction has no obvious efficiency in deoxygenating cellulose. Additionally, the O content of GP-225-0 cellulose was 49.7 wt.%, which was slightly lower than the raw and AP-225-0 cellulose. This implies that GP torrefaction realizes slight deoxidation of cellulose at a temperature as low as 225 °C. It is worth noting that the O content of GP-250-0 cellulose was 44.5 wt.%, which was even lower than that of AP-torrefied cellulose at 300 °C (48.8 wt.%) [10]. During the GP torrefaction at 250 °C, the O content of cellulose decreased continuously as the holding time extended, from 44.5 wt.% at 0 min to 27.2 wt.% at 60 min.

On the other hand, the C content of GP-torrefied cellulose increased with the holding time extension at 250 °C, reaching the highest content of 68.0 wt.% at 60 min. Moreover, the HHV of AP-torrefied cellulose was lower than 14.5 MJ/kg at 250 °C and 60 min, which was proximate to raw cellulose. Contrarily, the HHV of GP-torrefied cellulose sufficiently increased to as high as 25.0 MJ/kg at 250 °C and 60 min. The HHV of GP-250-60 torrefied cellulose was significantly higher than that of torrefied cellulose (16.8 MJ/kg) and hemicellulose (22.4 MJ/kg) by AP torrefaction at 300 °C and proximate to torrefied lignin (25.2 MJ/kg) by AP torrefaction at 300 °C [10]. Moreover, the HHV of GP-250-60 torrefied cellulose was higher than that of lignite (24.3 MJ/kg) and proximate to that of subbituminous coal (25.5 MJ/kg) [31]. The ultimate composition and calorific value confirm that the fuel properties of GP-250-60 cellulose are proximate to the subbituminous coal [31].

#### 2.2.2. Elemental Distribution

To understand the torrefaction process and mechanism, the investigation of O and C migration and transformation is crucial. The O removal rate is defined as the O transformation rate from raw cellulose to volatile, and the C recovery rate is defined as the C transformation rate from raw cellulose to torrefied cellulose in this work. The results are shown in Table 1. The O removal rates were as low as 3.2 wt.% by AP torrefaction at 250 °C and 60 min. Simultaneously, the C was almost completely retained in the AP-torrefied cellulose. These indicate that there was no obvious O and C migration and transformation during AP torrefaction. In other words, it is difficult for cellulose to thermally decompose during AP torrefaction at 200–250 °C. Nevertheless, the O and C migration and transformation phenomena were much more significant during GP torrefaction. The O removal rate increased with the temperature rise and holding time extension, reaching the highest O removal rate of 71.8 wt.% at 250 °C and 60 min. Meanwhile, the C recovery rate of GP-250-60 was still as high as 83.6 wt.%. This indicates that GP torrefaction realizes deeper deoxidation and C enrichment of cellulose under mild conditions.

The C/H/O molar ratios of torrefied cellulose were calculated through elemental composition and shown in Table 1. For the purpose of direct comparison with raw cellulose, the C molar ratio was expressed in the form of 6. The molar ratio of AP-torrefied cellulose had no obvious change compared to the raw cellulose with tiny amounts of H_2_O produced. This implies that the chemical structure of AP-torrefied cellulose had no significant change. During the GP torrefaction, much more volatile were produced, causing the C/H/O molar ratio of GP-torrefied cellulose to change significantly. The O molar ratio of GP-torrefied cellulose initially decreased during the GP torrefaction at 225 °C. Additionally, the molar ratios of O and H further decreased with the temperature rise and holding time extension, reaching the lowest molar ratio of 5.1 and 1.8 under 250 °C and 60 min, respectively. The molar ratios of H and O were much smaller than that of C, causing the GP-torrefied cellulose to be composed of aromatic C, which is completely different from raw cellulose and AP-torrefied cellulose.

The C/H/O molar ratio of volatile was obtained according to the ultimate composition difference between raw and torrefied cellulose. The C/H/O molar ratio of volatile is expressed in the form of CO_x_·yH_2_O. All the volatile components from AP torrefaction can be represented in the form of CO_2_ and H_2_O, and the ratios indicate they were mainly composed of H_2_O and a bit of CO_2_. The O molar ratios of volatile from GP torrefaction decreased significantly when the temperature reached 250 °C, indicating the CO- and O-containing organic molecules were generated during the GP torrefaction at 250 °C. Meanwhile, the H_2_O molar ratios continuously decreased with the holding time extension. This indicates that the O removal of cellulose is firstly in the form of CO_2_ and H_2_O and subsequently in the form of CO- and O-containing organic molecules during the GP torrefaction. This phenomenon and reactions were also observed during the GP torrefaction of LSW at 250 °C [29] and the pyrolysis of cellulose at 400–650 °C [32].

#### 2.2.3. Van Krevelen Diagram

**Figure 2:**
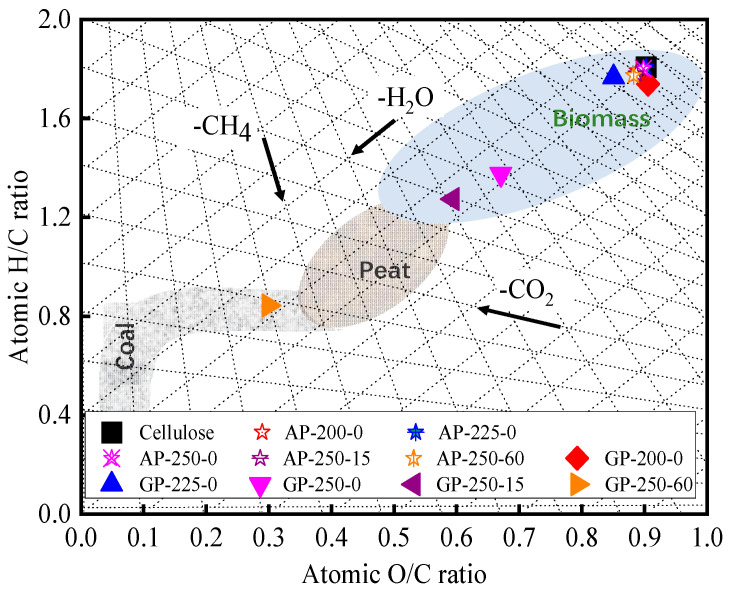
Van Krevelen diagram for raw and torrefied cellulose.

Figure 2 shows the O/C to H/C values of raw and AP- and GP-torrefied cellulose in the Van Krevelen diagram. It shows the fuel properties of AP-torrefied cellulose are close to the raw cellulose. The GP-200-0- and GP-225-0-torrefied cellulose are close to the raw cellulose, and the GP-250-0- and GP-250-15-torrefied cellulose are near the peat area. The GP-250-60-torrefited cellulose is located in the subbituminous coal area. Moreover, it also exhibits the deoxygenation pathway during torrefaction macroscopically. For the GP torrefactions from 200 °C to 250 °C, the main deoxygenation pathway is in the form of H_2_O removal. At the same time, there exists a clear CO_2_/CO removal trend for the GP torrefaction at 250 °C from 15 min to 60 min, which is consistent with the results shown in Table 1.

Furthermore, the chemical structures of raw and torrefied cellulose are highly related to their O/C and H/C ratios. The O/C and H/C ratios of raw, GP-200-0, and GP-225-0 cellulose were in the small range of 0.85–0.90 and 1.74–1.78, respectively. This indicates that their main chemical structures are similar to each other and composed of cyclic aliphatic compounds of glucose monomer [33]. The H/C ratio of GP-250-0 cellulose was as low as 1.38, indicating the aromatic nuclei structure had been generated [33]. The H/C ratio further decreased with the holding time extension, reaching the lowest value of 0.84 at 60 min. This implies that much more aromatic nuclei with branch aliphatic C structures were formed in torrefied cellulose. Thus, it can be concluded that the GP-torrefied cellulose is composed of an aromatic nucleus with branch aliphatic C through aromatization reaction during the GP torrefaction.

### 2.3. Chemical Structure

The product yields and elemental compositions of AP and GP torrefactions differ from each other greatly, indicating the thermal decomposition process and reaction mechanism are different for both torrefactions. AP torrefaction at 250 °C has almost no impact on cellulose decomposition and chemical structural evolution. Moreover, the thermal decomposition process and reaction mechanism of AP torrefaction have been widely studied. Therefore, the subsequent analyses only focus on the structural evolution of GP-torrefied cellulose in order to deeply understand its thermochemical process and mechanism.

#### 2.3.1. Crystal Structure

**Figure 3:**
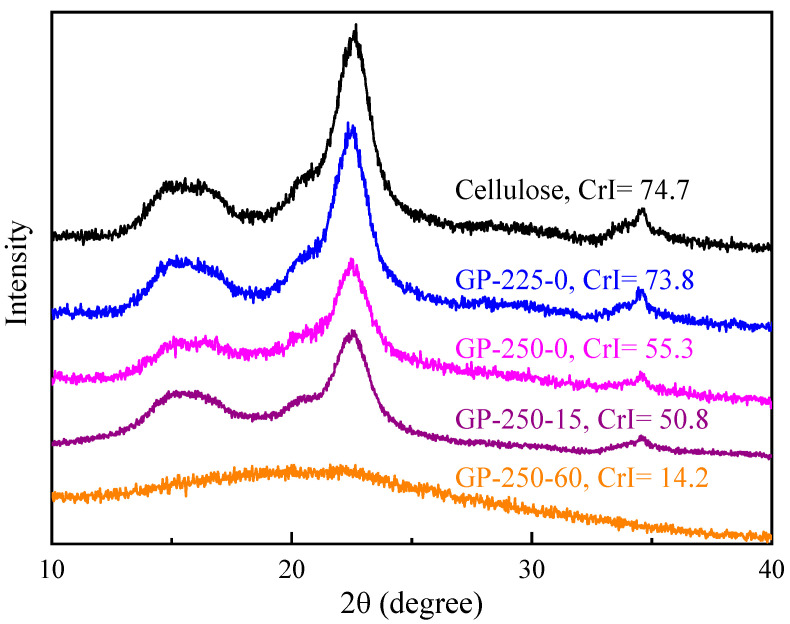
The XRD patterns of raw and GP-torrefied cellulose.

Figure 3 shows the XRD patterns and CrI values of raw and GP-torrefied cellulose. The CrI of GP-225-0 cellulose was 73.8%, which was slightly lower than that of raw cellulose at 74.7%. This indicates that a part of crystalline cellulose is depolymerized and converted into amorphous cellulose, causing a slight decrease in CrI value. During the GP torrefaction at 250 °C, the CrI value decreased with the holding time extension significantly, from 55.3% at 0 min to 50.8% at 15 min, reaching a value as low as 14.2% at 60 min, which is much lower than that of AP-torrefied cellulose at 200–300 °C [12]. This result implies that crystalline cellulose is largely thermally decomposed during GP torrefaction at temperatures as low as 250 °C.

#### 2.3.2. Organic Functional Group

**Figure 4:**
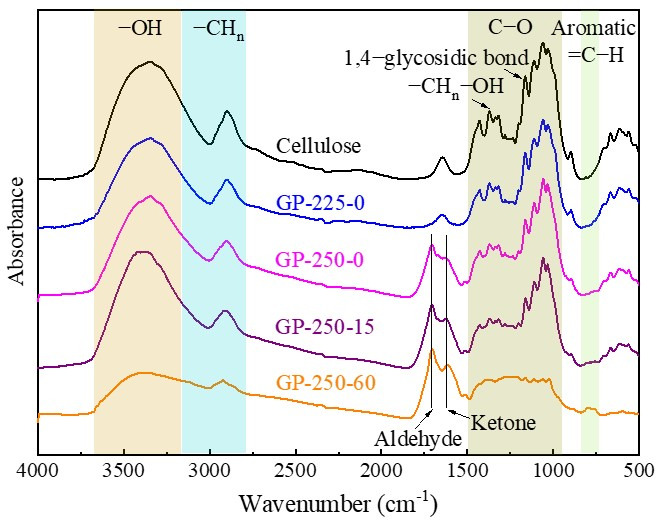
The FTIR spectrum of raw and GP-torrefied cellulose.

The FTIR spectrum was divided into six regions according to different types of functional groups, which are 3230–3500 cm^−1^ of -OH, 2877–3018 cm^−1^ of -CH_n_, 1705–1714 cm^−1^ of C=O in aldehydes, 1603–1620 cm^−1^ of C=O in ketones, 1000–1600 cm^−1^ of C-O, and 730–840 of =C-H in the aromatic ring [34], as shown in Figure 4. The functional groups in the raw cellulose were mainly composed of -OH, -CH_n_, and C-O. The functional group peak intensities of GP-225-0-torrefied cellulose showed no obvious change compared to the raw cellulose. The crystalline H_2_O (1560 cm^−1^), cellulose skeleton -CH_2_, -CH_n_-OH, and 1,4-glycosidic bond functional group intensities of GP-250-0-torrefied cellulose decreased. This indicates that the cellulose is depolymerized and the crystal structure is destroyed, which was also confirmed in the above discussion Section 2.3.1. Additionally, the continuous decrease in -OH peak intensity and enhancement in C=O peak intensity with torrefaction time extension at 250 °C imply that the inter- and intramolecular dehydration reactions occurred [35,36]. More importantly, the aromatic =C-H functional groups were generated through the aromatization reaction of cellulose during GP torrefaction at 250 °C. The GP-250-60-torrefied cellulose was mainly composed of aromatic =C-H in the aromatic ring, C=O in aldehydes and ketones, and -CH_n_.

#### 2.3.3. C Structure

**Figure 5:**
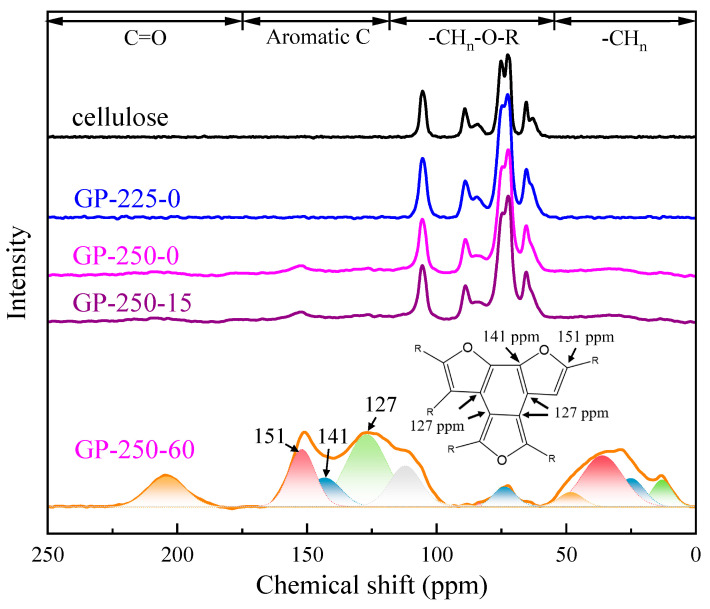
The ^13^C NMR of the raw and torrefied cellulose.

The structural evolution of torrefied cellulose was further investigated by ^13^C NMR, and the results are shown in Figure 5. The ^13^C NMR spectrum was divided into four regions according to different types of C functional groups, which are 0–50 of aliphatic C, 50–110 of O-alkyl C, 110–160 of aromatic C, and 160–230 ppm of carbonyl C functional groups. To accurately describe the C structure evolution of cellulose during torrefaction, the ^13^C NMR curves were divided into their corresponding C functional group by Gaussian peak-splitting [37,38,39].

The aliphatic C was divided into four peaks of 13, 25, 36, and 48 ppm vs. 109, 127, 141, and 151 ppm for aromatic C. Their relative contents (%) and absolute contents (content_raw_, %; raw cellulose basis) of C structures are illustrated in Table 2. The relative content was used to explore the composition and percentage of the C functional group in the torrefied cellulose. The content_raw_ was used to explore the transformation of the C structure during the torrefaction.

The C-containing functional groups in the raw cellulose mainly located in O-alkyl C region, which is the typical C structure distribution of crystallized and amorphous cellulose. The C structure of GP-225-0-torrefied cellulose had no obvious change compared to the raw cellulose. After the GP torrefaction at 250 °C, the C structure of torrefied cellulose changed significantly. The content_raw_ of O-alkyl C decreased with the holding time extension, from 42.9% of raw cellulose to as low as 1.5% of GP-250-60 cellulose. Contrarily, the content_raw_ of aliphatic C, aromatic C, and carbonyl C increased. These results show that most of O-alkyl C is converted to aromatic C, followed by aliphatic C and carbonyl C, and their highest contents_raw_ were 20.4%, 10.5%, and 3.4% after GP torrefaction at 250 °C and 60 min, respectively. This clearly indicates that the torrefied cellulose is composed of the aromatic nucleus with branch C, which is also confirmed by the Van Kvellen diagram and FTIR spectrum.

The chemical shift of 63 to 105 ppm peaks belongs to the C1-6 of crystallized and amorphous cellulose. It is worth noting that the content_raw_ of amorphous C6 increased after GP torrefaction at 225 °C, while the content_raw_ of other C structures decreased. This implies that the conversion of crystallized cellulose to amorphous cellulose is the initial stage of GP torrefaction, which is consistent with the XRD results. The chemical shift of 109–151 ppm peaks belongs to the aromatic C structure. It was found the aromatic structure of cellulose decomposed at 180–650 °C was a furans structure, which was discovered through detailed characterization [38] and reaction pathway study [32]. The abundant aromatic C structure in GP-torrefied cellulose indicates that the cellulose undergoes significant aromatization reaction during the GP torrefaction at 250 °C. Furthermore, the content_raw_ of aliphatic C increased with the temperature rise and holding time extension, reaching the highest content_raw_ of as high as 10.5% at 250 °C and 60 min. It is due to the removed O-containing functional groups, resulting in the conversion from -CH_n_-OH to -CH_2_ and -CH_3_.

### 2.4. Molecular Structure Determination

**Figure 6:**
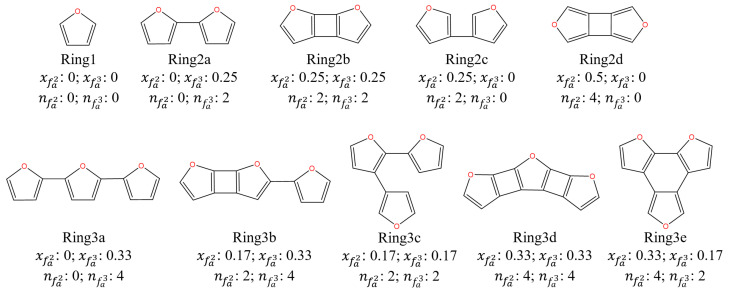
The skeleton structure of torrefied cellulose.

A structural parameter equation for the structure determination of intermediate and torrefied cellulose was established and shown in Table 3. In detail, the molecular structure determination and molecular structure establishment start with the confirmation of the C skeleton and branch structures. To facilitate, aromatic furans skeleton structures are listed in Figure 6. The furans skeleton of GP-torrefied cellulose contains two kinds of aromatic bridging C ($f_{a}^{2}$ and $f_{a}^{3}$), which are used to calculate the molar fraction of aromatic bridgehead carbon ($x_{f_{a}^{2}}\;and\;x_{f_{a}^{3}}$), respectively. The number of aromatic rings ($n_{ring}$) and aromatic C ($n_{ring-C}$) are then obtained. The numbers of $f_{a}^{2}\;$($n_{f_{a}^{2}}$) and ($n_{f_{a}^{3}}$) are further obtained according to their percentages in aromatic C through Equation (3). The C skeleton of torrefied cellulose is, thus, determined through the above numbers and the following two rules:

(1) The $x_{a}\;and\;x_{b}$ values correspond to a skeleton;
(2) The $n_{f_{a}^{2}}$ and $n_{f_{a}^{3}}$ values are consistent with the skeleton as further inspection criteria.

**Table 3:** Carbon structural parameters in GP-torrefied cellulose.

Structural Parameters	Symbol/Equations		Value		
			GP-250-0	GP-250-15	GP-250-60
Molar fraction of aromatic bridgehead carbon	$x_{f_{a}^{2}}=f_{a}^{2}/f_{a};x_{f_{a}^{3}}=f_{a}^{3}/f_{a}$	(1)	0.28; 0.25	0.17; 0.19	0.34; 0.20
Number of aromatic rings	$n_{ring}$		2	3	3
Number of aromatic C in aromatic ring	$n_{ring-C}=4\times n_{ring}$	(2)	8	12	12
Number of aromatic C in aromatic ring	$n_{f_{a}^{2}}=x_{f_{a}^{2}}\times n_{ring-C};n_{f_{a}^{3}}=x_{f_{a}^{3}}\times n_{ring-C}$	(3)	2; 2	2; 2	4; 2
Number of substituted branchs of aromatic ring	$n_{sub-C}=n_{ring-C}\times {(f}_{a}^{4})/f_{a}$	(4)	3	4	2
Number of terminal C in branch	$n_{tml-C}=n_{sub-C}$	(5)	3	4	2
Number of intermediate C in branch	$n_{int-C}=n_{sub-C}\times {(f}_{al}^{2}+f_{c}-f_{al}^{3})/f_{a}^{4}$	(6)	6	7	5
Number of terminal -CH_3_ in branch	$n_{tml-CH3}=n_{sub-C}\times f_{al}^{1}/f_{a}^{4}$	(7)	1	1	1
Number of terminal -CHO in branch	$n_{tml-CHO}=n_{sub-C}\times f_{al}^{3}/f_{a}^{4}$	(8)	2	3	1
Number of -CH_2_- in branch	$n_{int-CH2}=n_{sub-C}\times f_{al}^{2}/f_{a}^{4}$	(9)	4	5	4
Number of -CO- in branch	$n_{int-CO}=n_{sub-C}\times (f_{c}-f_{al}^{3})/f_{a}^{4}$	(10)	2	3	1
Structural equation			C_17_H_14_O_6_	C_23_H_18_O_9_	C_19_H_16_O_5_
C/H/O molar ratio			C_6_H_4.9_O_2.1_	C_6_H_4.7_O_2.4_	C_6_H_5.1_O_1.6_
Molecular structure schematic	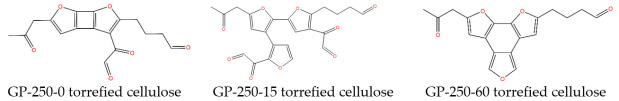				

For example, the $x_{a}$ and $x_{b}$ of GP-250-0 cellulose were 0.28 and 0.25. So, its C skeleton structure was ring2b and the values of $n_{ring}$ and $n_{ring-C}$ were 2 and 8. Its $n_{f_{a}^{2}}$ and $n_{f_{a}^{3}}$ values were 2 and 2, which confirmed the rationality of the C skeleton. Additionally, the branch structure contains terminal and intermediate C functional groups, such as -CH_3_, -CH_2_, -CH, and -CO-. The number of substituted branches of the skeleton ($n_{sub-C}$) is calculated by Equation (4) through the $f_{a}^{4}$ percentages in aromatic C. As we all know, the value of $n_{sub-C}$ equals $n_{tml-C}$ due to each branch having a corresponding terminal C functional group. Moreover, it is worth noting that a partial C=O structure forms a terminal aldehydes structure (-CHO) with a -CH structure. Other C=O structure forms ketones (-CO-) structure in the branch due to the limitation of the triple-coordination of C in the skeleton structures, resulting in the C in the skeleton structures not being able to directly bond with C=O to form a furan=C=O structure. So, the contents of $f_{al}^{3}$ and ($f_{c}-f_{al}^{3}$) also represent the aldehydes and ketones contents. So, the number of intermediate C in a branch is calculated by Equation (6) through the intermediate C percentages in $f_{a}^{4}$. The numbers of each terminal and intermediate C functional group are calculated by Equations (7)–(10) through their percentages. Thus, the molecular structure of torrefied cellulose can be confirmed based on the above division and calculation.

The C structure parameters of intermediate and GP-torrefied cellulose are illustrated in Table 3. It shows that the aromatic ring grew with the holding time extension from a two-ring at 0 min to a three-ring in 15 min during the GP torrefaction at 250 °C. This implies that the two-ring aromatic furans nucleus in GP-torrefied cellulose is formed rapidly during GP torrefied at 250 °C, followed by the formation of a three-ring through the polymerization reaction of furans. Additionally, the $n_{sub-C}$ first increased from 3 in GP-250-0 cellulose to 4 in GP-250-15 cellulose and then decreased to 2 in GP-250-60 cellulose. This indicates that the polymerization reaction of furans is accompanied by the removal of an unstable substituent, especially the conversion of branched ketone and aldehyde to CO. Additionally, the C/H/O molar ratios of intermediate and GP-torrefied cellulose were proximate to the C/H/O molar ratios obtained from elemental analysis, as shown in Table 1.

### 2.5. GP Torrefaction Mechanism of Cellulose

**Figure 7:**
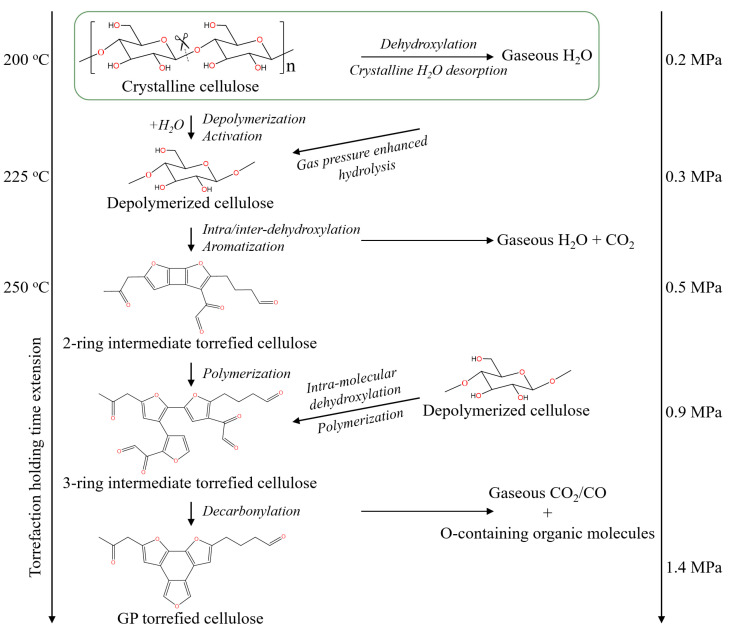
The schematic diagram of the GP torrefaction mechanism of cellulose.

The comprehensive GP torrefaction mechanism of cellulose is illustrated based on the results presented in this study, and it is shown in Figure 7. At the GP torrefaction temperature of 200 °C, crystalline H_2_O was desorbed to produce the initial gaseous H_2_O. Due to the GP torrefaction in a closed system, the generated volatile molecules are retained in the reactor. The secondary reactions, such as hydrolysis, between volatile molecules and cellulose in the closed system then occur [40]. Meanwhile, the volatiles raise the gas phase pressure in the reactor, and the raised pressure and increased chemical potential of volatile molecules further enhanced the secondary reactions greatly. So, the sufficiently enhanced secondary reactions cause the breakdown of the 1,4-glycosidic bond in cellulose and the formation of amorphous and active cellulose at temperatures as low as 200–225 °C.

At the temperature of 225–250 °C, the amorphous and active cellulose undergo significant aromatization reactions to form the two-ring furans aromatic compounds, and the removal of O-containing function groups is mainly through the production of H_2_O. As the holding time extension at 250 °C, the aromatic furans nucleus grows from a two-ring structure to a three-ring structure through the polymerization reaction of the furans polymer, which enhances the removal of the ketone and aldehyde function groups in intermediate torrefied cellulose and forms gaseous CO- and O-containing organic molecules. Thus, the formation mechanism of low O content-torrefied cellulose has been clearly understood.

## 3. Materials and Methods

### 3.1. Materials

The raw cellulose was commercial α-cellulose (Aladdin, Shanghai, China; CAS, 9004-34-6). The average molecular mass of cellulose was 162 g/mol, and its particle size was 250 μm. The C, H, and O mass percentages of raw cellulose on dry basis (d.b.) were 42.5, 6.4, and 51.1 wt.%, respectively.

The HHV of raw and torrefied cellulose was calculated through Dulong’s equation [41]. This was in order to directly compare with reported HHV of lignocellulose and its components. The HHV of raw cellulose was 14.4 MJ/kg.

### 3.2. Torrefaction Experiment

**Figure 8:**
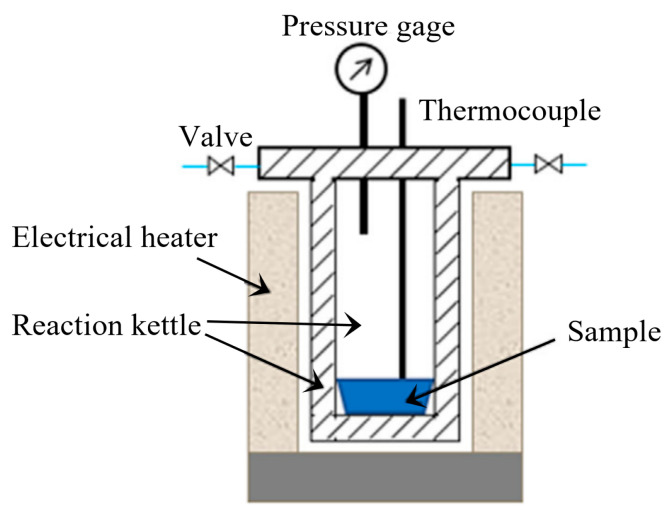
The schematic diagram of the GP torrefaction system.

As shown in Figure 8, the GP torrefaction system consists of reaction kettle, heating system, and temperature and pressure detectors. A total of 10 g cellulose was placed in the reaction kettle. The air was then removed from the torrefaction system by using N_2_ as carrier gas. During the GP torrefaction, the carrier gas was kept off throughout the experiment. The gas pressure rise was caused by the temperature increase and generated volatiles in the closed reaction kettle. The reaction was still heated from room temperature to 200, 225, and 250 °C at a heating rate of 5 °C/min and was maintained for 15 and 60 min at 250 °C. The final pressures for these five experiments were 0.2, 03, 0.5, 0.9, and 1.4 MPa, respectively.

After GP torrefaction, the solid product (torrefied cellulose) was collected in the reaction. The mass of solid product was measured by weighing. Additionally, for direct comparison purposes, the AP torrefaction was conducted through thermo gravimetric analysis (TGA, Woden, Australia, Germany-resistant, STA449F3) under the same condition. The experiments were named “method-temperature-time” according to GP/AP, temperatures, and holding time. To illustrate, the “GP-200-0” represents the GP torrefaction at 200 °C with a holding time of 0 min.

### 3.3. Characterization

Elemental analyzer (Elementar, Langenselbold, Germany, Vario CHNEL-2) was used to detect the mass percentages of C, H, and N in raw and torrefied cellulose. X-ray diffraction (XRD, Netherlands Pana Branch X’Pert PRO) was used to analyze the crystal morphology of raw and torrefied cellulose. The scanning rate was 4°/min in the 2θ range of 10–80°. The CrI was calculated by Equation (11) [42], where the I_002_ and I_am_ are the peaks of 22.5° and 18°, respectively.

CrI = (I_002_ − I_am_) × 100%/I_002_ (11)

The Fourier transform infrared spectroscopy (FTIR, Bruker Vertex 70) was used to analyze the composition of organic functional groups in raw and torrefied cellulose. The spectral scan range and resolution were 400–4000 cm^−1^ and 4 cm^−1^, respectively.

The cross-polarization/magic angle spinning (CP/MAS) ^13^C Nuclear Magnetic Resonance (NMR) was used to quantify the C structure in raw and torrefied cellulose. The resonance frequency and spin rates were 150.9 and 14 kHz, respectively. The contact time and cycle delay time were 3 ms and 5 s, respectively. The chemical shifts of ^13^C were externally referenced with (CH_3_)_4_Si (TMS).

## 4. Conclusions

Cellulose begins to thermally decompose during GP torrefaction at temperatures as low as 225 °C, and its fuel properties are enhanced with the temperature rise and holding time extension, reaching the highest HHV of as high as 25.0 MJ/kg at 250 °C and 60 min. At the same time, AP torrefaction at 200–250 °C has almost no impact on cellulose decomposition and its fuel property enhancement. A molecular structure calculation method was developed for the structure determination and reaction mechanism investigation of intermediate and torrefied cellulose. The intermediates are formed by intra/inter-molecular dehydroxylation reactions vs. polymerization reactions for GP-torrefied cellulose. A deoxygenation and aromatization mechanism model was then developed. This work provides theoretical guidance for the optimization of the gas-pressurized torrefaction method and a study method for the molecular-level structure determination and mechanism investigation of the thermal conversion processes of LSW.

## Figures and Tables

**Table 1:** The ultimate composition, O removal rate, C recovery rate, and higher heating value (HHV) of raw AP- and GP-torrefied cellulose; C/H/O molar ratio of volatile and torrefied cellulose.

Samples	Ultimate Composition (wt.%, d.a.f.)			HHV (MJ/kg)	O Removal Rate (wt.%)	C Recovery Rate (wt.%)	C/H/O Molar Ratio of Volatile	C/H/O Molar Ratio of Torrefied Cellulose
	C	H	O ^a^					
Cellulose	42.5	6.4	51.1	14.4	/	/	/	C_6_H_10.8_O_5.4_
AP-200-0	42.9	6.4	50.8	14.6	0.0	100.0	/	C_6_H_10.8_O_5.3_
GP-200-0	42.9	6.3	50.8	14.5	0.8	100.0	/	C_6_H_10.7_O_5.3_
AP-225-0	42.5	6.4	51.1	14.4	0.0	100.0	CO_2.0_·57.8H_2_O	C_6_H_10.8_O_5.4_
GP-225-0	43.8	6.5	49.7	15.2	6.6	98.8	CO_1.9_·3.2H_2_O	C_6_H_10.6_O_5.1_
AP-250-0	42.6	6.4	51.0	14.4	0.5	100.0	CO_2.0_·40.0H_2_O	C_6_H_10.8_O_5.4_
GP-250-0	49.8	5.7	44.5	17.0	29.4	95.0	CO_0.3_·5.0H_2_O	C_6_H_8.2_O_4.0_
AP-250-15	42.9	6.4	50.7	14.5	1.8	99.9	CO_2.0_·21.7H_2_O	C_6_H_10.7_O_5.3_
GP-250-15	52.7	5.6	41.7	18.4	38.0	93.4	CO_0.6_·4.5H_2_O	C_6_H_7.6_O_3.6_
AP-250-60	42.9	6.3	50.4	14.5	3.2	99.0	CO_2.0·_17.9H_2_O	C_6_H_10.6_O_5.3_
GP-250-60	68.0	4.8	27.2	25.0	71.9	83.6	CO_0.7_·3.3H_2_O	C_6_H_5.1_O_1.8_

^a^: Calculated by difference.

**Table 2:** The relative content and content_raw_ (%) of C in raw and torrefied cellulose.

Chemical Shift	C Structure	Symbol	Relative Content (%)					Content_raw_ (%)				
			Cellulose	GP-225-0	GP-250-0	GP-250-15	GP-250-60	Cellulose	GP-225-0	GP-250-0	GP-250-15	GP-250-60
0–60	Aliphatic C	$f_{al}$	0	0	6.9	7.8	29.5			2.8	3.1	10.5
13	-CH_3_	$f_{al}^{1}$			1.3	1.40	3.9			0.5	0.6	1.4
25–36	-CH_2_	$f_{al}^{2}$			4.0	4.00	20.7			1.6	1.6	7.4
48	-CH	$f_{al}^{3}$			1.6	2.40	4.9			0.6	1.0	1.7
60–110	O-alkyl C	$f_{ao}$	100	100	82.2	76.9	4.2	42.9	41.9	33.2	30.8	1.5
63	Amorphous C6		5.1	6.6	3	3.00	0.1	2.2	2.8	1.2	1.2	0.0
66	Crystalline C6		8.8	8.7	8.1	8.60	0.3	3.8	3.6	3.3	3.4	0.1
74	C2,3,5		54.4	53.6	47	42.00	2.6	23.3	22.5	19.0	16.8	0.9
84	Amorphous C4		5.7	5.8	2.5	2.70	0.2	2.4	2.4	1.0	1.1	0.1
89	Crystalline C4		9.7	9.1	9	8.50	0.2	4.2	3.8	3.6	3.4	0.1
105	C1		16.3	16.3	12.6	12.1	0.8	7.0	6.8	5.1	4.8	0.3
110–165	Aromatic C	$f_{a}$	0	0	7.8	10.5	56.9			3.2	4.2	20.4
109	C-H	$f_{a}^{1}$			0.8	2.90	14.2			0.3	1.2	5.1
127	C-C	$f_{a}^{2}$			2.1	1.80	18.6			0.8	0.7	6.7
141	O-C=C	$f_{a}^{3}$			1.9	2.00	13.8			0.8	0.8	4.9
151	C-CHxR	$f_{a}^{4}$			3	3.80	10.3			1.2	1.5	3.7
165–235	Carbonyl C	$f_{c}$	0	0	3.1	4.80	9.5			1.3	1.9	3.4
204	C=O	$f_{c}$			3.1	4.80	9.5			1.3	1.9	3.4
Total		$f_{total}$	100	100	100.0	100	100	42.9	42.0	40.4	40.0	35.8

## Data Availability

Data will be made available upon request.
